# Impact of a Precision Intervention for Vascular Health in Middle-Aged and Older Postmenopausal Women Using Polar Heart Rate Sensors: A 24-Week RCT Study Based on the New Compilation of Tai Chi (Bafa Wubu)

**DOI:** 10.3390/s24175832

**Published:** 2024-09-08

**Authors:** Xiaona Wang, Yanli Han, Haojie Li, Xin Wang, Guixian Wang

**Affiliations:** 1 Chinese WuShu Academy, Beijing Sport University, Beijing 100084, China; bsuzgwsxy@163.com (X.W.); 2019210192@bsu.edu.cn (Y.H.); 2417@bsu.edu.cn (G.W.); 2 School of Exercise and Health, Shanghai University of Sport, Shanghai 200483, China; 3 Physical Education College, Zhengzhou University, Zhengzhou 451000, China

**Keywords:** Tai Chi (BaFa WuBu), heart rate sensor, precision intervention, menopausal women, vascular health

## Abstract

(1) Background: This study utilized a 24-week intervention incorporating heart rate sensors for real-time monitoring of intervention training, aiming to comprehensively assess the effects of Tai Chi on vascular endothelial function, atherosclerosis progression, and lipid metabolism. The insights gained may inform personalized non-pharmacological interventions to enhance the management of cardiovascular health in this population to provide sustainable benefits and improve quality of life. (2) Methods: Forty postmenopausal middle-aged and elderly women were randomly assigned to an exercise or control group. The exercise group underwent a 24-week Tai Chi (BaFa WuBu) training intervention with real-time heart rate monitoring using Polar sensors. Pre- and post-intervention assessments included body composition, blood pressure, vascularity, and blood parameters measured with the Inbody 720, Vascular Endothelial Function Detector, and Arteriosclerosis. Data were analyzed using SPSS 26.0 and mixed-design ANOVA to assess the effects of time, group, and their interactions on study outcomes. (3) Results: After training through 24 weeks of Tai Chi (BaFa WuBu) intervention, compared with the control group, systolic blood pressure in the exercise group was significantly lower (*p* < 0.05), and the difference between left and right arm pulse pressure, left and right ankle mean arterial pressure, left and right side baPWV, left and right side ABI, TC, TG, LDL, and blood pressure viscosity were all very significantly lower (*p* < 0.01), and the diastolic blood pressure was significantly higher (*p* < 0.05). Compared with baseline values in the exercise group, systolic blood pressure, right and left arm pulse pressure difference, right and left ankle mean arterial pressure, right and left side baPWV, right and left side ABI, TC, TG, LDL, and blood pressure viscosity decreased very significantly (*p* < 0.01) and diastolic blood pressure and FMD increased very significantly (*p* < 0.01) in the exercise group after the intervention. (4) Conclusions: In our study, a 24-week Tai Chi (BaFa WuBu) program significantly improved vascular health in middle-aged and older postmenopausal women. This simplified Tai Chi form is gentle and effective, ideal for older adults. Regular practice led to reduced vascular obstruction, improved lipid metabolism, and enhanced vascular endothelial function, crucial for preventing vascular diseases. The real-time heart rate sensors used were pivotal, enabling precise monitoring and adjustment of exercise intensity, thereby enhancing the study’s scientific rigor and supporting Tai Chi (BaFa WuBu) as a beneficial therapeutic exercise.

## 1. Introduction

Cardiovascular Disease (CVD), which includes atherosclerosis (AS), hypertension, and myocardial infarction, poses significant morbidity risks among middle-aged and elderly populations [1]. Aging leads to systemic organ and cellular decline, with pronounced effects on the cardiovascular system. Key manifestations of cardiovascular aging include myocardial remodeling, reduced cardiac function, atherosclerosis, and heightened endothelial damage [2,3]. Epidemiological data indicate that CVD prevalence is lower in premenopausal women compared to men of similar age, but rises notably post-menopause, potentially surpassing male risks [4,5].

Postmenopausal women face heightened cardiovascular disease (CVD) risks primarily due to declining estrogen levels, as indicated by Framingham Heart Study risk scores [6]. Estrogen plays a crucial role in cardiovascular protection, and its reduction correlates closely with conditions like hypertension and atherosclerosis [7]. Studies underscore that menopause significantly increases atherosclerosis risks compared to men of similar age [8]. Understanding these mechanisms is crucial for preventing CVD in this demographic [9,10]. Additionally, decreased endothelial function in menopausal women poses another critical cardiovascular risk factor, influencing abnormal vascular tone and platelet activity [11]. Estrogen’s protective role in endothelial function is highlighted, with its absence contributing to atherosclerosis and thrombosis [12]. Moreover, changes in blood lipid metabolism during menopause, such as decreased high-density lipoproteins (HDL) and increased low-density lipoproteins (LDL) and triglycerides, significantly impact cardiovascular health and atherosclerosis progression [13,14]. Non-pharmacological interventions, particularly aerobic exercise, emerge as pivotal for mitigating these risks. Research shows aerobic exercise effectively slows atherosclerosis progression, improves blood vessel elasticity, and reduces cardiovascular event risk [15]. Regular exercise also lowers cholesterol levels and enhances vascular endothelial function, thereby reducing arterial inflammation [16,17]. Studies suggest that expending 1000 kcal weekly through exercise can reduce cardiovascular mortality by 20% over five years [18]. Even single moderate-intensity exercise sessions have been shown to improve endothelial function, while biweekly sessions over several months reduce inflammatory markers and enhance vascular function in older adults [19,20]. Aerobic exercise achieves these benefits by enhancing nitric oxide synthase activity, thereby improving blood flow and balancing oxidative stress [21,22]. However, challenges such as joint strain and fracture risks from high-intensity exercise must be considered, especially for menopausal women with poorer physical conditions [23]. In conclusion, while aerobic exercise offers significant cardiovascular benefits for postmenopausal women, tailored approaches are necessary to ensure safety and sustained participation in exercise routines.

Tai Chi, as a low-intensity, low-load, easy-to-learn, and easy-to-practice aerobic exercise, shows potential advantages in cardiovascular health interventions for menopausal women [24]. Through slow and smooth movements, Tai Chi helps to improve cardiovascular function and promote blood circulation, and compared with other high-intensity exercises, Tai Chi puts less pressure on joints and bones, which is suitable for the physiological characteristics of menopausal women [25]. For older adults, traditional forms of Tai Chi can be complicated, so simplifying and personalizing Tai Chi exercises can be positive for specific populations. An example of a simpler and easier-to-learn introductory Tai Chi routine is BaFa WuBu, introduced by the General Administration of Sport of China (GASC), which is more concise and saves time and energy compared to traditional Tai Chi.

In addition, previous studies have demonstrated the vital role that heart rate sensors play in exercise. By monitoring the heart rate variability of individuals in real time, we are able to gain a deeper understanding of the impact of exercise on the body and its effects. Studies have shown that heart rate patterns during exercise can reflect the body’s adaptation to exercise and endurance level, which is crucial for developing a personalized exercise program [26]. In terms of exercise monitoring, heart rate sensors can not only provide heart rate data during exercise, but also analyze indicators such as heart rate variability, which are important for assessing post-exercise recovery and body adaptation. Real-time monitoring further enhances the understanding of physiological responses during exercise, allowing us to adjust the intensity and mode of exercise in a timely manner to achieve better training results and safety [27]. Real-time monitoring is even more critical in exercise interventions. By continuously monitoring the heart rate changes of the exerciser, we are able to accurately assess the effectiveness of the intervention and its impact on the body. This personalized real-time feedback not only improves the relevance and effectiveness of the intervention, but also helps to optimize the exercise program so that it is more closely aligned with the individual’s health needs and goals [28]. Studies have shown that by monitoring heart rate variability during exercise, it is possible to assess the adaptations of the cardiovascular system and infer an individual’s level of aerobic capacity. This can help athletes and coaches to better develop training programs to maximize exercise effectiveness and health benefits [29]. In addition, in the medical field, heart rate sensors are widely used to monitor a patient’s response to exercise and its impact on treatment outcomes. For example, it has been found that in cardiovascular rehabilitation, by monitoring heart rate and heart rate variability in real time, rehabilitation programs can be adjusted more accurately, thus improving patients’ rehabilitation outcomes and quality of life [30]. And studies in the elderly population have shown that regular aerobic exercise can significantly improve cardiac health and overall physiologic status. These studies, which utilized heart rate sensors for continuous monitoring, found that exercise was effective in reducing the risk of cardiovascular disease and helped maintain functional independence and quality of life in older adults [31]. In summary, the application of heart rate sensors in exercise not only helps to gain a deeper understanding of the body’s physiological responses, but also improves the precision and real-time nature of exercise monitoring, thus providing a scientific basis and technical support for personalized exercise interventions.

In summary, previous studies on the therapeutic effects of tai chi on middle-aged and older menopausal women have been less well researched and have had shorter intervention cycles. In contrast, our study will utilize a 24-week intervention cycle combined with heart rate sensors to monitor subjects’ heart rate and other physiological indicators to accurately assess the long-term effects of Tai Chi on cardiovascular health in menopausal women. Through this precise study design, we hope to gain a comprehensive understanding of the potential benefits of Tai Chi (BaFa WuBu) as a sustainable non-pharmacological intervention program in the management of cardiovascular health in middle-aged and elderly women. Our study aims to delve into the effects of Tai Chi on various aspects of vascular endothelial function, degree of atherosclerosis, and lipid metabolism, and to provide a scientific basis for the development of a more personalized and effective intervention program.

## 2. Participants and Method

### 2.1. Participants

Prior to the commencement of the experiment, the required sample size was pre-estimated by using the G*Power 3.1 software to ensure that the experiment had sufficient test validity. The test yielded β = 0.82 (β > 0.8 indicates a valid sample size), as well as a significance level α set at 0.05 [32], which was calculated to point out that the total sample size required for this study was 36 participants. Therefore, 40 healthy postmenopausal women were selected for this study and randomly assigned to two groups, i.e., the exercise group (EG) and the control group (CG), each with 20 participants, by means of a computer-generated randomization sequence. The exercise group underwent a 24-week training intervention of Tai Chi (BaFa WuBu), and the control group underwent a routine of an unregulated exercise workout as per their daily routine.

Subject inclusion criteria: (1) those without carotid atherosclerotic plaques; (2) all subjects were questioned about their medical history to confirm the absence of cardiovascular disease, diabetes mellitus, hypertension, hyperlipidemia, malignant tumors, infectious or inflammatory diseases, etc., in order to avoid the involvement of disease in influencing the function of vascular endothelial cells and to reduce the influence of irrelevant variables; (3) no cardiovascular disease-related medications were taken in the past 3 months. Before the experiment, all subjects (Table 1) had their basic information counted and understood the process of exercise intervention in this experiment, participated in the experiment voluntarily, and signed an informed consent form. The study was approved by the Ethics Committee of Beijing Sport University (Ethics No. 2023294H) and complied with the Declaration of Helsinki.

### 2.2. Method

#### 2.2.1. Polar Heart Rate Sensor

**Figure 1:**
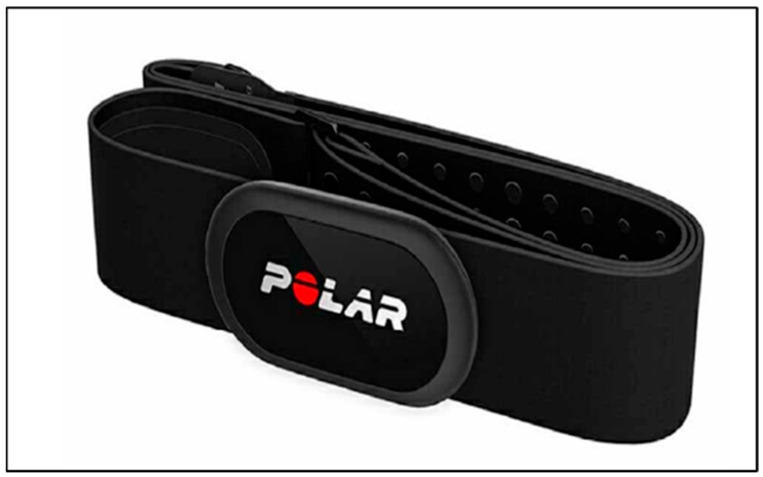
Polar heart rate sensor.

In this study, the Polar H10 chest strap heart rate sensor from Polar Company of Finland is used, which has the following main features: equipped with a comfortable, lightweight, soft elastic bandage, easy to remove for cleaning; wireless analog heart rate signal, the transmission frequency is 5 kHz, the effective transmission distance is up to 80 cm; the newly launched product adopts the digital code encryption technology, the transmission frequency is 2.4 GHz, the effective transmission distance is up to 3 m; equipped with a button type lithium battery, the working life is more than 2000 h; it has excellent anti-exercise interference characteristics, suitable for sports requiring a certain amplitude and jumping; the front locking buckle design makes it possible to use Polar H10 heart rate sensor. The new product adopts digital coding encryption technology, the transmission frequency is 2.4 GHz, and the effective transmission distance is up to 3 m; equipped with a button-type lithium battery, the working life is more than 2000 h; with excellent anti-interference characteristics of the movement, it is suitable for the movement that requires a certain amplitude and jumping; the front lock design makes the sensor’s installation and uninstallation operation fast and easy (Figure 1).

Therefore, this sensor is particularly suitable for elderly motion monitoring applications. In this study, real-time monitoring was used to ensure that the subjects’ heart rate was stabilized within 60% of their maximum heart rate. Previous studies have shown that keeping the heart rate at about 60% of the maximum heart rate during exercise in older adults ensures both safety and good exercise results [33]. Therefore, this study utilizes heart rate sensors for real-time monitoring, aiming to effectively and accurately intervene and guide the exercise of older adults.

#### 2.2.2. Experimental Control

During the exercise, the exercise intensity was controlled by combining the subjective feelings of the subjects (RPE11-14). Subjects were asked about their subjective fatigue during the breaks between each group, and were controlled by observing the amplitude of movement, the degree of force, the respiratory rate, and the facial condition to ensure that the subjects were able to withstand the intensity of the exercise at this stage of teaching or training. If the exercise intensity is greater than the subjective tolerance, then reduce the exercise density and increase the interval time; finally, at the end of each training session, ask and record the subject’s fatigue and physical recovery after training, if there is cumulative fatigue or greater intensity, then take the initiative to reduce the amount of exercise, reduce the time of exercise or the number of practice sessions, gradual and safety-oriented.

#### 2.2.3. 24-Week Tai Chi (BaFa WuBu) Training Intervention Program

The 24-week Tai Chi (BaFa WuBu) intervention in the exercise group was preceded by a one-week acclimatization training period for all subjects in the exercise group, which was completed under the guidance of a national Tai Chi athlete during the acclimatization period. Subjects’ heart rates were monitored using an exercise bracelet for both the acclimatization training and the formal intervention. In this study, Tai Chi (BaFa WuBu) was mainly used for the intervention training, a 24-week training intervention, four training sessions per week, one one-hour session, and the training intervention was mainly divided into four phases: the basic phase (BP)—the improvement phase (IP)—the consolidation phase (CP)—the proficiency phase (PP), and the specific content was arranged (Table 2).

#### 2.2.4. Basic Indexes and Blood Indexes Testing

The following indicators were tested in the morning before and after the exercise intervention: (1) body mass index (BMI) and body weight (BMI) were tested by Inbody 720 body composition meter; (2) blood glucose, blood lipid, and plasma viscosity tests: subjects were asked to fast 12 h in advance, and 30 mL of fasting venous blood was taken in the morning at regular intervals, and whole blood was used for blood glucose test after venous blood collection. The serum was centrifuged at 3000 rpm within 2 h, and the test indexes included total cholesterol (TC), triglyceride (TG), high-density lipoprotein cholesterol (HDL-C), and low-density lipoprotein cholesterol (LDL-C). Plasma viscosity was measured after centrifugation of blood collected with anticoagulation tubes. Blood samples for each index were taken 48 h after exercise.

#### 2.2.5. Vascular Function Test

(1) Blood pressure testing of the extremities

After lying down for 10 minutes (min) at room temperature, a special cuff was wrapped around each extremity (the lower edge of the cuff was 2–3 cm from the transverse elbow stripe in the upper extremity and 2 cm from the ankle in the lower extremity), and a cuff of approximately 2.4 cm was wrapped around the metatarsal bone of the first toe of the patient.

(2) Vascular endothelial function (brachial FMD) test

The endothelial-dependent flow-mediated endothelial diastolic function of the brachial artery was tested using a vascular endothelial function tester (UNEX EF, Tokyo, Japan), and the right arm was measured uniformly. After measurement of the basal diameter, the cuff was pressurized for 5 min and then released, and the blood flow shear stress generated by the increase in reflex blood flow was applied to the vessel wall, and the endothelial function was inferred from the measurement of the rate of change of the internal diameter of the vessel. Subjects were asked to fast for 4 h before the test, refrain from smoking, alcohol, caffeine, or antioxidant drugs for 12 h, and rest for at least 10 min in a quiet room at a comfortable temperature before the test.

(3) Brachial ankle pulse wave conduction velocity (baPWV) test

Currently, baPWV is used to reflect the elasticity of the large arteries and the middle arterial system, and the increase suggests a decrease in the elasticity of the blood vessels of the whole body. baPWV is measured by the oscillometric technique, and the test instrument is the Omron BP-203R PE2 atherosclerosis tester of Japan. baPWV is calculated by measuring the distance between the two recorded parts of the brachial artery and the ankle artery, where the pulse is more pronounced (L), and pulse wave conduction time (t), and then by calculating the formula baPWV. The formula baPWV (mm/s) = L/t is used. l is the distance between the two detectors, and t is the time difference between the two waveforms. baPWV is generally calculated by recording 16 consecutive values of baPWV, discarding the 3 maximum and 3 minimum values, and taking the average of the 10 measurements. An increase in the value of baPWV suggests that arterial elasticity is decreasing, and arteriosclerosis occurs. baPWV is normally in the range of 800 to 1400. Normal values range from 800 to 1400 mm/s.

(4) Ankle Brachial Index (ABI) Test

The ABI reflects the possibility of blockage of peripheral arteries and is an independent predictor of cardiovascular and cerebrovascular events and mortality, and is negatively correlated with mortality. The subject lies flat on the bed, and the ankle blood pressure of the posterior tibial artery and the dorsalis pedis artery is measured by wrapping a blood pressure cuff around the ankle, and the ratio of its blood pressure to that of the upper arm is the ankle–brachial blood pressure ratio, i.e., ABI = ankle systolic blood pressure value/upper arm systolic blood pressure value. Normal value is 0.9–1.3, ABI < 0.5 suggests the existence of multiple arterial obstructions; 0.5 < ABI < 0.8 suggests the existence of one arterial obstruction; 0.8 < ABI < 0.9 suggests the possibility of arterial obstruction; ABI > 1.3 suggests vascular calcification.

#### 2.2.6. Data Statistics

SPSS 26.0 software was used to statistically analyze all the test data and the results were expressed as mean ± standard deviation, to find out the mean and standard deviation of each group in the pre- and post-test. Subsequently, a mixed-design ANOVA was used to explore whether the time factor, the group factor, and their interactions had a significant effect on the test results. A setting of *p* < 0.05 indicates that this result is statistically different.

## 3. Results

### 3.1. Changes in Blood Pressure-Related Indices in Middle-Aged and Elderly Postmenopausal Women before and after 24 Weeks of Tai Chi (BaFa WuBu) Intervention

**Figure 2:**
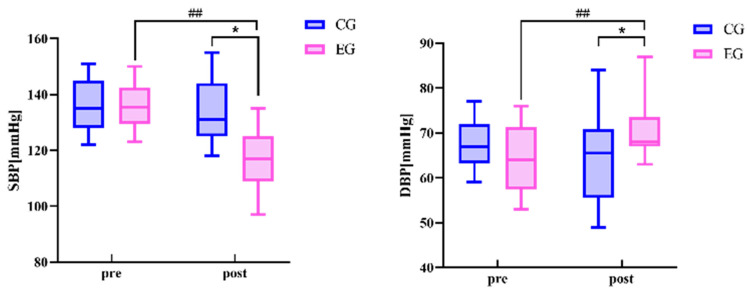
Effects of 24 weeks of Tai Chi (BaFa WuBu) intervention on systolic and diastolic blood pressure in middle-aged and elderly postmenopausal women, with the control group in blue and the exercise group in pink.

**Figure 3:**
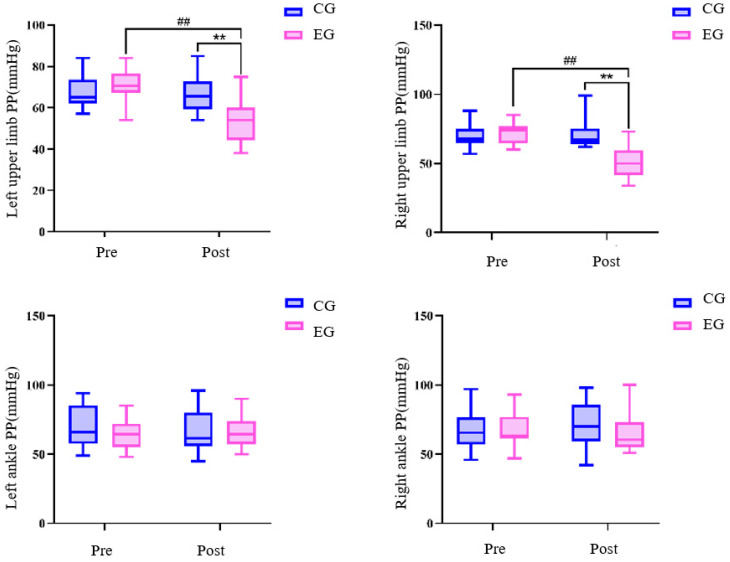
Effects of 24 weeks of Tai Chi (BaFa WuBu) intervention on pulse pressure difference in middle-aged and elderly postmenopausal women, control group in blue and exercise group in pink.

**Figure 4:**
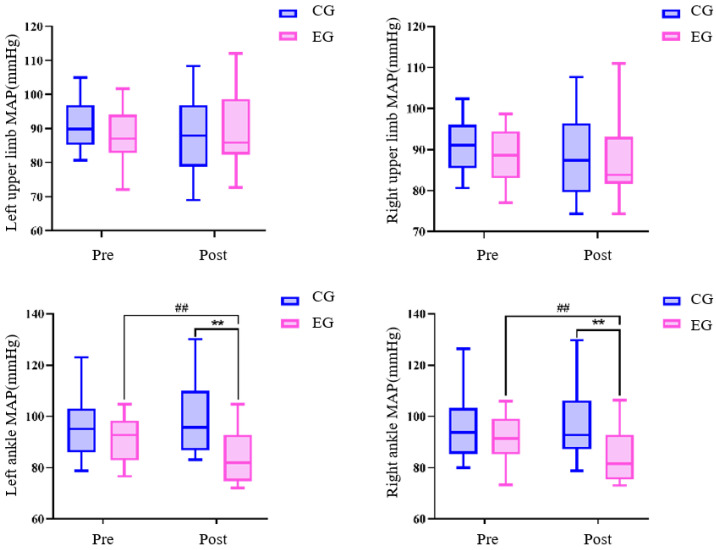
Effects of 24 weeks of Tai Chi (BaFa WuBu) intervention on mean arterial pressure in middle-aged and elderly postmenopausal women, blue is control group, pink is exercise group.

There was no difference in all variables between the exercise group and the control group before the exercise intervention (Table 3 and Figure 2, Figure 3 and Figure 4) (* *p* < 0.05, ** *p* < 0.01, # *p* < 0.05, ## *p* < 0.01 for all figures). Systolic blood pressure, pulse pressure difference (left arm), pulse pressure difference (right arm), mean arterial pressure (left ankle), and mean arterial pressure (right ankle) in the exercise group after the 24-week Tai Chi (BaFa WuBu) intervention saw very significant decreases compared to the pre-intervention period (*p* < 0.01), and also very significant decreases compared to the control group after the intervention (*p* < 0.01), and systolic blood pressure was a significant decrease in the control group (*p* < 0.01), and mean arterial pressure was a significant decrease in the control group (*p* < 0.01), and systolic blood pressure was a significant decrease (*p* < 0.05). Diastolic blood pressure in the exercise group increased very significantly (*p* < 0.01) compared to pre-intervention and significantly (*p* < 0.05) compared to the control group. There was no significant change in all variables before and after the intervention in the control group.

### 3.2. Changes in Vascular Function in Middle-Aged and Elderly Postmenopausal Women before and after 24 Weeks of Tai Chi (BaFa WuBu) Intervention

**Figure 5:**
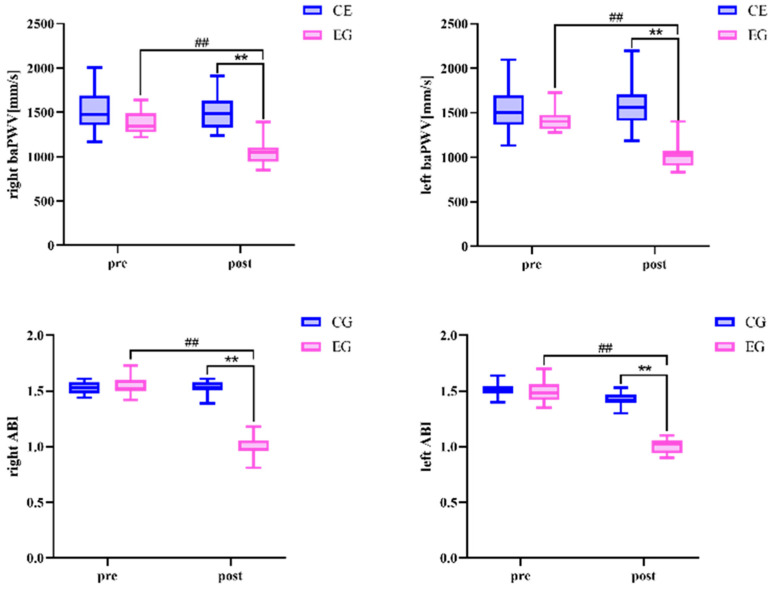
Effects of 24 weeks of Tai Chi (BaFa WuBu) intervention on vascular stiffness in middle-aged and older postmenopausal women. baPWV is the arm–ankle pulse wave conduction velocity and ABI is the ankle–brachial index; blue is the control group and pink is the exercise group.

**Figure 6:**
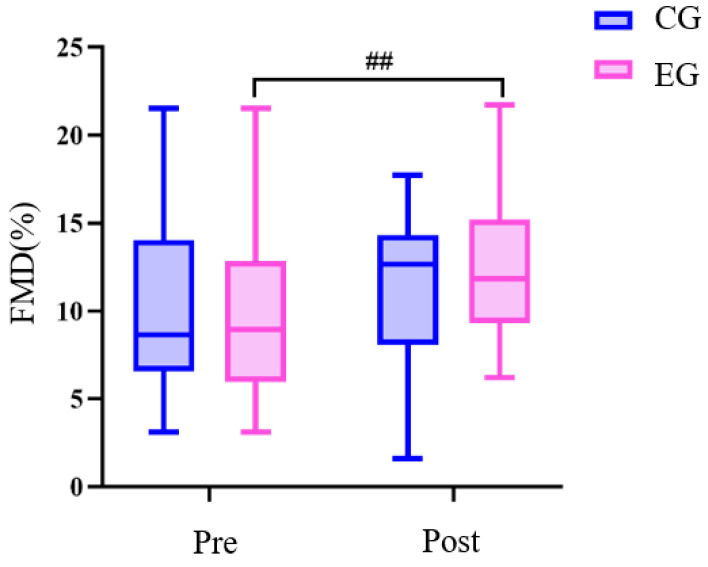
Effects of 24 weeks of Tai Chi (BaFa WuBu) intervention on vascular endothelial function in middle-aged and older postmenopausal women, FMD is brachial artery flow-mediated vasodilatation function; control group in blue, exercise group in pink.

There was no difference in all variables between the exercise group and the control group before the exercise intervention (Table 4 and Figure 5 and Figure 6). Left baPWV, right baPWV, left ABI, and right ABI in the exercise group after the 24-week Tai Chi (BaFa WuBu) intervention decreased significantly compared to the pre-intervention period (*p* < 0.01), and compared to the control group (*p* < 0.01). FMD increased very significantly after the intervention (*p* < 0.01) and did not change significantly compared to the control group. There was no significant change in all variables before and after the intervention in the control group.

### 3.3. Changes in Blood Indices in Middle-Aged and Elderly Postmenopausal Women before and after 24 Weeks of Tai Chi (BaFa WuBu) Intervention

**Figure 7:**
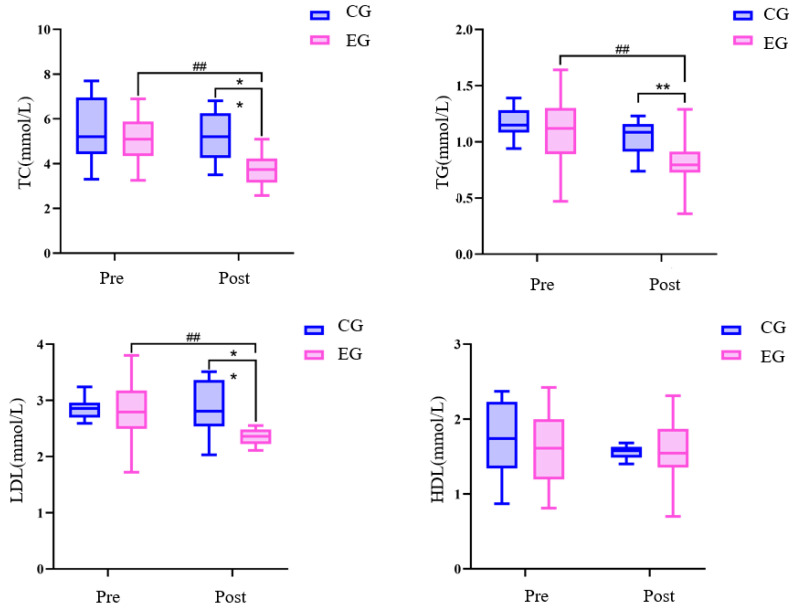
Effects of 24 weeks of Tai Chi (BaFa WuBu) intervention on lipids in middle-aged and older postmenopausal women; TC is total cholesterol, TG is triglyceride, LDL is low-density lipoprotein, and HDL is high-density lipoprotein; blue is the control group and pink is the exercise group.

**Figure 8:**
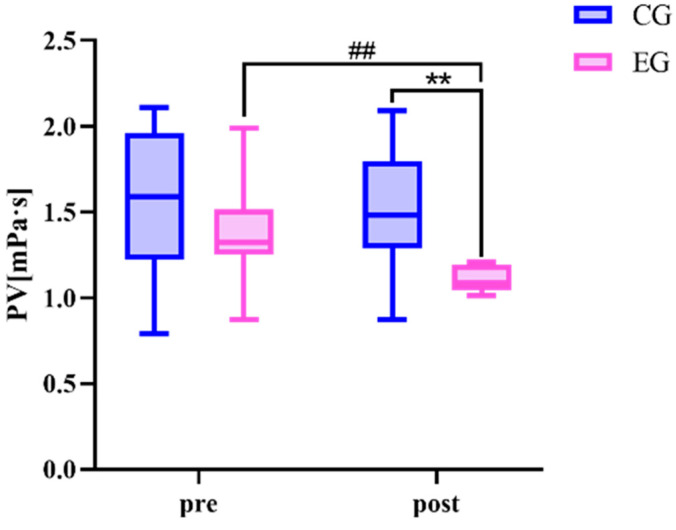
Effect of 24 weeks of Tai Chi (BaFa WuBu) intervention on plasma viscosity in middle-aged and older postmenopausal women, control group in blue and exercise group in pink.

There was no difference in all variables between the exercise group and the control group before the exercise intervention (Table 5 and Figure 7 and Figure 8). The 24-week Tai Chi (BaFa WuBu) intervention resulted in a significant decrease in TC, TG, LDL, and plasma viscosity in the exercise group compared to the pre-intervention period (*p* < 0.01), and also compared to the control group (*p* < 0.01). There was no significant change in all variables before and after the intervention in the control group.

## 4. Discussion

### 4.1. Effect of 24-Week Tai Chi (BaFa WuBu) Intervention on Blood Pressure in Menopausal Women

In this study, we found that a 24-week (BaFa WuBu) intervention resulted in a significant decrease in systolic blood pressure and a significant increase in diastolic blood pressure in postmenopausal women. The decrease in systolic blood pressure was consistent with the study of Cebula et al. [34], where 6 weeks of Nordic walking resulted in a significant decrease in systolic blood pressure within the normal range of values in normotensive postmenopausal women, suggesting a significant improvement in arterial vascular elasticity and systolic function, which may be attributed to the fact that long-term regular aerobic training interventions induce an increase in the bioavailability of NO, which in turn leads to an improvement in the NO-dependent vasodilatory function [35,36,37]. However, in this paper, we found that diastolic blood pressure increased significantly after 24 weeks of Tai Chi (BaFa WuBu) training intervention, which was different from previous studies, which showed a significant increase in diastolic blood pressure after the intervention, from the lowest critical value of 64.25 ± 7.22 mmHg to 71.15 ± 6.83 mmHg. Protogerou et al. [38] reported that in order to avoid the risk of low diastolic blood pressure, the optimal diastolic blood pressure in middle-aged and elderly people should be about 70 mmHg, and when diastolic blood pressure is less than 60 mmHg, it negatively affects coronary perfusion during the diastolic phase of the heart, which in turn triggers heart failure and leads to reduced survival. Therefore, the 24-week Tai Chi (BaFa WuBu) intervention positively affected vascular function in postmenopausal women, resulting in optimal blood pressure levels, which was not found in previous studies and is the first finding of this study.

In addition, previous studies have noted that excessive pulse pressure significantly elevates the risk of cardiovascular accidents [39,40]. In the present study, we found that 24 weeks of Tai Chi (BaFa WuBu) intervention resulted in a significant reduction in pulse pressure in the left and right arms of postmenopausal women and a return from abnormal levels to within the normal range, indicating significant improvement in the structure, elasticity, and vascular function of the arterial vascular wall. This is consistent with the findings of Patil et al. [41] that 2 months of traditional yoga exercise significantly improved pulse pressure in the elderly. There was no significant change in pulse pressure at the ankle, but in combination with the index of mean arterial pressure at the ankle, it is evident that the vascular adaptation of the lower limbs was improved, and it is possible that such a level of pulse pressure at the ankle is appropriate for this subject group.

Mean arterial pressure reflects cardiac function and resistance of the peripheral large arteries. In the present study, it was found that 24 weeks of Tai Chi (BaFa WuBu) intervention resulted in a significant improvement in mean arterial pressure at the left and right ankle in postmenopausal women, with a reduction of approximately 8 mm Hg on both sides. In agreement with the study of Zarins et al. [42], 5 weeks of cycling aerobics intervention resulted in a reduction in mean arterial pressure by approximately 8 mm Hg and a significant improvement in cardiovascular adaptations in postmenopausal women. This in turn induced a significant reduction in ankle mean arterial pressure. Mean arterial pressure in the upper extremities did not change significantly due to the fact that systolic blood pressure in the upper extremities was significantly reduced after the training intervention; however, diastolic blood pressure was elevated to an optimal level, so overall mean blood pressure did not change, but vascular adaptations in the upper extremities were similarly improved.

In addition, this paper found a significant negative trend of diastolic blood pressure correlation with vascular blockage (ABI) and triglycerides before and after the intervention; this is due to the fact that diastolic blood pressure increased significantly to the optimal range after the 24-week Tai Chi (BaFa WuBu) intervention, which in turn was able to effectively promote vascular and lipid health, which is in keeping with Farron’s [43] study, which concluded that exercise significantly improves diastolic blood pressure and lipid metabolism in hypertensive patients. This was demonstrated by an improvement in blood pressure into the normal range, resulting in improved vascular health.

### 4.2. Effects of a 24-Week Tai Chi Intervention Program on Vascular Function in Menopausal Women

In addition, this experiment further explored Tai Chi (BaFa WuBu) to improve vascular endothelial function in middle-aged and elderly menopausal women. The initial stage of atherosclerosis manifests as vascular endothelial dysfunction. The results of this study showed that 24 weeks of Tai Chi (BaFa WuBu) intervention significantly elevated FMD in healthy menopausal women, indicating a significant improvement in vascular endothelial function. Numerous studies have shown that endothelial dysfunction associated with aging can be remodeled through regular participation in physical activity [44,45,46], mainly by lowering the body’s blood pressure, blood glucose, and lipid levels, and improving neurohormone release. The results of the study in this paper also show this, where after 24 weeks of Tai Chi (BaFa WuBu) intervention, the training exercise group’s ABI status, triglycerides, and LDL showed a positive correlation downward trend, and the improvement in lipid health greatly contributed to the state of vascular health.

Arterial stiffness responds to the dilatory properties of the arterial vascular system, and better elasticity protects arteries and maintains blood flow [47]. The results of this study showed that baPWV and ABI were significantly decreased within normal values after 24 weeks of Tai Chi (BaFa WuBu) intervention, and arterial vascular elasticity was significantly improved in healthy postmenopausal women. Ho [48] et al. reported that 8 weeks of interval sprint training resulted in a significant decrease of 7.2% in baPWV and a significant increase of 14% in aerobic fitness in postmenopausal women. Others found that 20 min of aerobic exercise per day resulted in a 6.2% decrease in baPWV in postmenopausal women. Barone et al. [49] reported that a 6-month intervention of aerobic combined with strength training in 140 middle-aged and elderly subjects improved the ABI of 15% of the individuals to the 1.0–1.3 range. It has also been reported that 12 months of aerobic exercise resulted in a significant reduction in ABI values within the normal range in healthy menopausal women [50], all of which remain consistent with the results of this study.

### 4.3. Effects of a 24-Week Tai Chi Intervention Program on Vascular-Related Risk Factors in Menopausal Women

Abnormal lipid metabolism in humans is a major predisposing factor for atherosclerosis and atherosclerotic plaque formation, and therefore lipid levels are considered to be important risk factors for vascular endothelial dysfunction [51]. This study further revealed the positive effects of Tai Chi (BaFa WuBu) on lipid metabolism in menopausal women. Significant decreases in total cholesterol (TC), triglyceride (TG), and low-density lipoprotein cholesterol (LDL) levels were observed during the 24-week intervention of Tai Chi, while plasma viscosity was also significantly reduced. This finding is consistent with the findings of Li NC [52] et al. who concluded that 12 weeks of aerobic exercise induced a decrease in HDL levels, TG, TC, and LDL levels in menopausal women to improve the lipid metabolism of the organism. In addition, Christos et al. [53] found that 16 weeks of aerobic training significantly increased HDL-C by 17.2% and decreased TG by 18.9% in postmenopausal women to improve the lipid metabolic status of the organism. The results of this study are consistent with previous studies on aerobic exercise on lipid metabolism, further supporting Tai Chi (BaFa WuBu) as an effective exercise intervention. Taken together, these findings provide a scientific basis for the use of Tai Chi (BaFa WuBu) as an alternative or adjunctive therapy to promote cardiovascular health in menopausal women, and they provide an important reference for further related research and clinical practice.

## 5. Limitations and Perspectives

First, the relatively small sample size of this study may affect the broad applicability of the findings. Second, this study did not assess the long-term sustained effects of the Tai Chi intervention and lacked long-term follow-up data, particularly in terms of sustained improvements in health status and quality of life at the end of the intervention. Further, this study only used Tai Chi as an intervention and did not compare other forms of exercise or non-pharmacological interventions. Future studies need to explore the differences between different exercise or non-pharmacological interventions and their relative strengths and weaknesses. Finally, because the study population was healthy menopausal women, the results may not be directly generalizable to groups with cardiovascular disease or other health problems, which limits the understanding and dissemination of its broader application. Future studies need to include more diverse groups, especially individuals who already have CVD or other health risks, to assess the generalizability and effectiveness of Tai Chi as a sustainable, nonpharmacological intervention.

## 6. Conclusions

By providing a 24-week Tai Chi (BaFa WuBu) training intervention to middle-aged and older postmenopausal women, we found that the program significantly improved participants’ vascular health. Tai Chi (BaFa WuBu), a newly simplified form of Tai Chi Chuan, is a gentle and effective training method that is particularly suitable for older adults and other special populations to practice and is easy to learn. Through the long-term practice of Tai Chi, we observed its ability to significantly reduce vascular obstruction and atherosclerosis, improve lipid metabolism, and effectively promote vascular endothelial function, thus having a positive impact on the prevention and amelioration of vascular diseases.

In addition, we particularly emphasize the importance of the heart rate sensor used in this study to monitor the exercise intervention in real time. The use of sensors allowed us to precisely monitor and adjust the exercise status and heart rate response of each participant, thereby increasing the accuracy and credibility of the intervention effect. This technological innovation not only enhances the scientific validity of the study, but also provides scientific support and theoretical foundation for Tai Chi (BaFa WuBu) as an effective exercise therapy.

## Figures and Tables

**Table 1:** Subjects’ basic information table.

Groups	Age (Year)	Height (cm)	Weight (kg)	Years of Menopause (Year)
EG	54.2 ± 4.60	161 ± 7.61	62.61 ± 11.33	4.7 ± 1.30
CG	53.7 ± 4.26	160.4 ± 4.56	56.6 ± 6.43	4.8 ± 1.73
	BMI (kg/m^2^)	Pulse (beat)	Blood glucose (mmol/L)	Physical activity level MET (in/week)
EG	23.8 ± 3.00	75.4 ± 10.2	5.23 ± 0.46	510.0 ± 67.43
CG	22.0 ± 2.23	74.3 ± 7.44	5.53 ± 0.38	490.0 ± 54.78

Note: (EG): exercise group; (CG): control group.

**Table 2:** The 24-week Tai Chi (BaFa WuBu) training intervention program.

Phases	Week	Preparatory Segment (10 min)	Basic Part (40 min)	Closing Segment (10 min)
BP	1–6	Perform breathing exercises, gentle swinging of the limbs, and simple Tai Chi footwork exercises	Learn the eight techniques, Peng, Lv, Ji (pressing), An, Cai, Lie (laying), Zhou, and Kao. Learn the five steps, Jin, Tui, Gu, Pan, and Ding	Stretching exercises: shoulder stretching horizontal, shoulder stretching vertical, leg stretching
IP	7–12	Incorporate more diversified Tai Chi basic steps and body flexibility exercises	Training the eight techniques, Peng, Lv, Ji (pressing), An, Cai, Lie (laying), Zhou, Kao. Training the five steps, Jin, Tui, Gu, Pan, and Ding Train the integrity of the maneuvers and footwork movements, and train the complete (BaFA WuBu) Eight Methods and Five Steps in a full set.	Stretching exercises: shoulder stretching horizontal, shoulder stretching vertical, leg stretching
CP	13–18	Introducing aids such as Tai Chi softballs to warm up and increase body flexibility and strength	The fluidity and expression of the inner strength of Tai Chi Quan are enhanced by practicing and correcting Tai Chi (BaFa WuBu) movements to improve the coherence of the overall routine.	Stretching exercises: shoulder stretching horizontal, shoulder stretching vertical, leg stretching
PP	19–24	The internal work of Tai Chi is trained mainly through the staking of Tai Chi	Enhance proficiency in Tai Chi (BaFa WuBu) complete routines.	Stretching exercises: shoulder stretching horizontal, shoulder stretching vertical, leg stretching

Note: Four phases: the basic phase (BP)—the improvement phase (IP)—the consolidation phase (CP)—the proficiency phase (PP). Eight techniques of Tai Chi (BaFa WuBu): Peng (warding off), Lv (rolling back), Ji (pressing), An (pushing), Cai (plucking), Lie (laying), Zhou (elbowing), Kao (leaning sideways). Five steps of Tai Chi (BaFa WuBu): Jin (advancing), Tui (retreating), Gu (shifting left), Pan (shifting right), and Ding (central equilibrium).

**Table 3:** Changes in blood pressure-related indices in middle-aged and elderly postmenopausal women after 24 weeks of Tai Chi (BaFa WuBu) intervention.

Indicators (Unit: mmHg)		CG		EG	
		Pre-Testing	Post-Test	Pre-Testing	Post-Test
SBP		137.70 ± 10.97	135.00 ± 12.71	136.10 ± 8.17	122.55 ± 16.25 *##
DBP		67.40 ± 5.28	64.35 ± 9.37	64.25 ± 7.22	71.15 ± 6.83 *##
PP	Left arm	68.05 ± 7.84	66.40 ± 7.81	70.95 ± 7.98	54.45 ± 10.32 **##
	Right arm	70.30 ± 7.61	70.65 ± 9.22	71.85 ± 7.38	51.40 ± 11.26 **##
	Left ankle	69.75 ± 14.19	67.05 ± 14.94	64.65 ± 11.52	65.75 ± 10.91
	Right ankle	68.35 ± 14.17	71.55 ± 16.03	66.90 ± 12.29	65.05 ± 13.85
MAP	Left arm	91.08 ± 7.26	87.78 ± 10.90	87.45 ± 7.94	89.55 ± 9.68
	Right arm	90.83 ± 6.76	87.90 ± 9.67	88.20 ± 6.70	88.28 ± 9.54
	Left ankle	95.05 ± 11.02	98.95 ± 13.12	91.10 ± 7.97	84.17 ± 10.02 **##
	Right ankle	94.83 ± 11.63	97.25 ± 12.53	91.75 ± 9.21	84.68 ± 10.42 **##

Note: SBP is systolic blood pressure, DBP is diastolic blood pressure, PP is Pulse pressure, MAP is mean arterial pressure. ## *p* < 0.01 indicates a highly significant change; * is a between-group comparison, * *p* < 0.05 indicates a significant change, ** *p* < 0.01 indicates a highly significant change.

**Table 4:** Changes in vascular function in middle-aged and older postmenopausal women with 24 weeks of Tai Chi (BaFa WuBu) intervention.

Unit	Indicators ()	CG		EG	
		Pre-Testing	Post-Test	Pre-Testing	Post-Test
mm/s	baPWV (Right)	1512.45 ± 210.77	1496.55 ± 188.17	1377.05 ± 119.59	1051.55 ± 138.79 **##
mm/s	baPWV (Left)	1530.75 ± 232.27	1592.45 ± 252.64	1415.85 ± 111.46	1030.75 ± 155.71 **##
\	ABI (Right)	1.53 ± 0.05	1.53 ± 0.05	1.55 ± 0.07	0.99 ± 0.08 **##
\	ABI (Left)	1.51 ± 0.05	1.50 ± 0.09	1.43 ± 0.05	1.01 ± 0.06 **##
%	FMD	10.92 ± 5.76	10.87 ± 4.31	9.59 ± 4.59	12.17 ± 3.93 #

Note: baPWV is Brachial ankle pulse wave conduction velocity, ABI is ankle–brachial index, FMD is endothelium-dependent flow-mediated dilatation. # is a within-group comparison, # *p* < 0.05 indicates a significant change, ## *p* < 0.01 indicates a highly significant change; ** *p* < 0.01 indicates a highly significant change.

**Table 5:** Effects of 24 weeks of Tai Chi (BaFa WuBu) intervention on blood lipids in middle-aged and elderly postmenopausal women.

Unit	Indicators	CG		EG	
		Pre-Testing	Post-Test	Pre-Testing	Post-Test
mmol/L	TC	5.50 ± 1.40	5.22 ± 1.04	5.14 ± 1.04	3.74 ± 0.76 **##
mmol/L	TG	1.17 ± 0.12	1.04 ± 0.15	1.06 ± 0.30	0.81 ± 0.22 **##
mmol/L	LDL	2.85 ± 0.16	2.86 ± 0.51	2.81 ± 0.57	2.35 ± 0.14 **##
mmol/L	HDL	1.76 ± 0.47	1.56 ± 0.08	1.58 ± 0.49	1.59 ± 0.42
mPa·s	PV	1.56 ± 0.42	1.54 ± 0.32	1.38 ± 0.25	1.11 ± 0.07 **##

Note: TC is total cholesterol, TG is triglyceride, HDL is high-density lipoprotein cholesterol, LDL is low-density lipoprotein cholesterol, PV is plasmic viscosity. ## *p* < 0.01 indicates a highly significant change; ** *p* < 0.01 indicates a highly significant change.

## Data Availability

The data that support the findings of this study are available on request from the corresponding author.
